# The Treatment of Acute Rheumatism and Its Complications

**Published:** 1892-12-10

**Authors:** 


### NORTHAMPTON GENERAL INFIRMARY.

### The Treatment of Acute Rheumatism and Its Complications.

Immediately on admission a patient with acute rheumatism is clothed in a flannel shirt, and placed in a bed in which sheets are dispensed with. The preliminary bath is omitted, and such washing as is absolutely necessary is done with as little exposure as possible. A diet of milk and beef tea is ordered, the temperature taken, and salicin in 10-grain doses is given to Dr. Buszard's patients, salicylate of soda to Dr. Jones's. In the case of the latter drug, if the temperature be considerably raised, 30 or 40 gr. are administered in carraway or camphor water, with a little spirit of chloroform, every two or three hours. In less acute cases 20 to 30 gr. are given every three or four hours. This is continued till the temperature has fallen below 100 deg. Fahr., and remains there. By lessening the dose or frequency, or both, the salicylate is reduced to 15 gr. three times a day. When the temperature has continued at normal (or, at any rate, not above 90 deg. Fahr. at night) for two days, the diet is advanced, provided also the joint pain and swelling and the perspiration have subsided. If there is no relapse, white fish (boiled) is ordered two days later, fowl two days later still, and after a similar interval chops and other butchers' meat. On the slightest indication of relapse return is made to the slop diet, and the dose or frequency of administration of salicylate of soda is augmented according to the severity of the relapse, but only exceptionally to the original dose. Two days after meat diet has been adopted the patient is allowed to get up a little if the state of the heart permit, and at the same time the salicylate is replaced by the ammonio citrate or other vegetable salt of iron. Where soda salicylate produces too much depression the ammonium salt is substituted, but occasionally the salicylates are of necessity entirely replaced by quinine, in doses of two grains, every three or four hours. The reduction of the quantity of the drug taken is also adopted earlier and more rapidly in such cases as show other toxic effects. Stimulant is not ordered as a rule at all, but for weak pulse, or where marked toxic effects are produced by salicylates or salicin, brandy is used.

Local treatment is not frequently adopted in subacute cases, but in the acute forms the joints are bandaged with cotton wool, and where the pain and tenderness are very severe blisters are occasionally used. In other cases where the staple treatment fails to relieve the pain, Dover's powder is given in the form of a pill. Chronic pain and swelling of the joints after the subsidence of the acute symptoms are treated by application of a solution of iodine and iodide of potassium, half as strong again as the tr. iodi, and swathing the joint in cotton wool and bandage. Gentle exercise is encouraged. Massage is used where the stiffness is very marked.

The heart is kept under daily observation till the temperature has been at normal for some days. The appearance of a rub is immediately dealt with by the application of a blister. Effusion is similarly treated, paracentesis being kept in reserve for grave cases resisting other treatment. An endocardial murmur appearing during the febrile stage is dealt with by prolonging the use of salicylate, or reducing it less rapidly. Should any indication exist of endocardial mischief, or myocarditis after the acute symptoms have subsided, the period of rest in bed is prolonged, strychnia is added to the tonic, and these cautions are still maintained till the second sound over the inner end of the second left intercostal space, the character of the first sound at the apex and the state of the pulse indicate that the heart can maintain its equilibrium.

The onset of pleurisy is dealt with by a sinapism. Effusion is first treated with a blister; failing this, a mercurial—pulv. hyd. subchlor gr. iij—iv., or pil. hydr. gr. iij—is administered, and if, in a week or ten days, the fluid still remains and is considerable in quantity, paracentesis is performed by means of an aspirator.

The temperature in ordinary cases is taken twice a day, but should 105 deg. Fahr. be reached, it is taken every hour or oftener, and if it continues to rise, cold sponging is adopted. A screen is placed round the patient, or he is removed to a small ward. The bedding is protected by a large waterproof sheet, with a layer of flannel or blanket immediately under the body, which is stripped of all clothing. The patient is sponged, at first with tepid, afterwards with cold, and in some cases with ice-cold water, the temperature being watched all the time. The reduction is not carried below 100 deg. Fahr., to allow for the fall which follows the sponging. In cases where the tendency of the temperature to rise is very great, the wet pack is alternated with the cold sponging. After a considerable reduction in temperature has been effected by the cold sponging a wet sheet is placed round the patient, wrapped round with the waterproof, and then with blankets, care being taken to avoid any raising of the head. This is continued, if necessary, for half an hour to three-quarters of an hour, when either the patient is dried and placed in dry blankets, or cold sponging is again resorted to. Antipyrin and other antipyretics are not used. Quinine in large doses is, however, employed before the temperature reaches 105 deg. Fahr., as well as during the hyperpyrexia.

